# Quality assessment of hydroquinone, mercury, and arsenic in skin-lightening cosmetics marketed in Ilorin, Nigeria

**DOI:** 10.1038/s41598-023-47160-2

**Published:** 2023-11-28

**Authors:** Olasunkanmi David Bamidele, Blessing Ayomide Kayode, Oluwasegun Ibrahim Eniayewu, Adebanjo Jonathan Adegbola, Raphael Segun Olatoye, Ngaitad Stanislaus Njinga, Sa’ad Toyin Abdullahi, Moji Taibat Bakare-Odunola

**Affiliations:** 1 Department of Pharmaceutical and Medicinal Chemistry, Faculty of Pharmaceutical Sciences, University of Ilorin, Ilorin, Nigeria; 2 School of Medical and Health Sciences, Edith Cowan University, Perth, Australia; 3 Department of Pharmaceutical Chemistry, Faculty of Pharmacy, Obafemi Awolowo University, Ile-Ife, Nigeria; 4 Department of Biochemistry, College of Basic Medical Sciences, University of Ibadan, Ibadan, Nigeria; 5 Department of Biochemistry, Faculty of Pure and Applied Sciences, Kwara State University, Malete, Nigeria

**Keywords:** Environmental chemistry, Chemical safety

## Abstract

Hydroquinone, Mercury (Hg), and Arsenic (As) are hazardous to health upon long-term exposure. Hydroquinone, Hg, and As were analysed in skin-lightening cosmetics randomly purchased from different cosmetic outlets within the Ilorin metropolis, Nigeria. The amount of hydroquinone in the samples was determined using a UV-spectrophotometry method at 290 nm. Hg and As were quantified using atomic absorption spectrophotometry (AAS). UV-spectrophotometry method validation showed excellent linearity (r^2^ = 0.9993), with limits of detection (0.75 µg/mL), limits of quantification (2.28 µg/mL), relative standard deviation (0.01–0.35%), and recovery (95.85–103.56%) in the concentration range of 5–50 µg/mL. Similarly, r^2^, LOD, and LOQ for Hg and As were 0.9983 and 0.9991, (0.5 and 1.0 µg/L) and 1.65 and 3.3 µg/L) respectively. All the samples contained hydroquinone, Hg and As in varying amounts. The amounts of hydroquinone, Hg and As present were in the ranges of 1.9–3.3%, 0.08–2.52 µg/g and 0.07–5.30 µg/g respectively. Only three of the analysed samples contained hydroquinone within the permissible limit of 2.0% w/w in cosmetic products. All the samples analysed contained mercury and arsenic in varying amounts. The need to periodically monitor the levels of hydroquinone, mercury, and arsenic in skin-lightening cosmetics marketed in Nigeria is recommended.

## Introduction

Globally, women, young girls, and some men frequently lighten their skin tones^1–3^. The skin-lightening industry is experiencing rapid growth on a global scale, driven by a substantial demand for cosmetics aimed at achieving lighter skin tones^2^. The use of skin-lightening cosmetics transcends sociodemographic boundaries^4,5^, including religious (Christianity, Islam, traditional beliefs), marital status (single, married, divorced), economic status (affluent and underprivileged), educational attainment (literate or illiterate), and social class (low, middle, and upper)^3–5^.The overwhelming desire for skin-lightening cosmetics in society is mostly due to the idea that having light skin is a sign of beauty, success, and power^5^. Some other reasons given for using skin-lightening cosmetics include improving personal appearance, following current fashion trends, treating skin imperfections like acne or melasma, refining facial and bodily complexion, and satisfying the preferences of one’s spouse^4,5^.

Nigeria, other African countries, and Asians, demonstrate a significant consumer base for skin-lightening cosmetics. This trend is corroborated by the WHO survey that reported 77% of Nigerian women frequently use skin-lightening cosmetics, as opposed to 25, 27, 35, and 59% for Mali, Senegal, South Africa, and Togo, respectively^6,7^. Many of these skin-lightening cosmetics, which are regrettably smuggled into Nigeria, contain banned substances, including hydroquinone and heavy metals. Many other countries around the world also use skin-lightening cosmetics^8,9^. Nevertheless, a significant concern arises from the inclusion of hazardous substances such as hydroquinone, mercury (Hg), and arsenic (As) in many of these cosmetics^10–19^. Hydroquinone, Hg, and As have all been identified as potentially dangerous chemicals in skin-lightening cosmetics^10–19^. While these chemicals are hazardous and cause major health problems, they can be sold illegally or under alternative brand names and are frequently not disclosed on the label^4^.

Hydroquinone is an aromatic organic compound and a potent inhibitor of melanin production used in the treatment of melasma^9,20^. Melasma, pigmented acne scars, post-inflammatory hyperpigmentation, and skin discoloration are major disorders of hyperpigmentation^21^, and the topical preparations for treating this disorder include hydroquinone, kojic acid, azelaic acid, glycolic acid, retinoids, and salicylic acid^21^. Specifically, hydroquinone remains the most effective lightening agent for treating common hyperpigmentation disorders globally^21^. Tyrosinase, a rate-limiting enzyme in the conversion of tyrosine to melanin in human skin, is inhibited by hydroquinone^21–23^. This inhibition decreases the production of melanin in the melanocytes, which explains its ability to lighten the skin^21–23^. Although it is a widely used skin-lightening agent, it is characterised by several adverse effects, which include carcinoma^4,9,24^, irritative dermatitis, melanocyte destruction, contact dermatitis, and ochronosis^21,25^, that leave the skin discoloured when consistently applied for a long period of time^4,26–28^. The hydroquinone metabolites p-benzoquinone and glutathione conjugates are frequently linked to the development of cancer^18,24,25^. When hydroquinone-containing cosmetics are used topically over an extended period, p-benzoquinone and hydroquinone conjugates with glutathione may accumulate and cause DNA damage and mutations^21^.

Hydroquinone-induced ochronosis adverse effects have been reported to be more prevalent among Africans who use skin-lightening cosmetics. For instance, 756 of the 789 cases of exogenous ochronosis reported in a study were of African extraction^10,25^. Concerns over the safety issues surrounding the use of hydroquinone have led many countries around the world, including the UK, EU, US, Australia, Asia, Africa, and others^9,10,29,30^ to prohibit the use of hydroquinone in cosmetics^28,29^. For instance, in 1980, the government of South Africa set a 2% permissible limit on hydroquinone in cosmetics^4^. Similarly, the US, UK, and EU instituted regulations with a 2% limit for cosmetic products and a 4% limit for topical preparations intended for dermatological use^5,8,9,23,25^. In Nigeria, the National Agency for Food and Drug Administration and Control (NAFDAC) initially authorised a limit of 2% hydroquinone in skin-lightening cosmetics^4^. The adverse effects of hydroquinone in cosmetics persist despite the limit. Consequently, the use of hydroquinone in cosmetics was eventually banned. Nonetheless, cosmetics containing hydroquinone continue to be used freely, with many lacking ingredient labelling, manufacturer addresses, and NAFDAC registrations^4^. Generally, ochronosis was noted even with cosmetic formulations containing 2% hydroquinone when used for an extended period^4^. In addition to the pharmaceutical uses of hydroquinone, it has been extensively mishandled by the cosmetics industry in unregulated skin-lightening cosmetics, causing safety issues with significant public health relevance. Most often, these ingredients are intentionally added by the manufacturers in amounts exceeding the permissible limit, primarily to increase the sales of their cosmetic products^20,31^. Although many countries globally have banned the use of hydroquinone in skin-lightening cosmetics, where its use is permitted, it should not exceed 2%^28^.

Prolonged and excessive exposure to mercury (Hg) and arsenic (As) has been shown to result in their accumulation in the body, disrupting normal body functions. Specifically, toxicants in cosmetics cause skin damage such as irritation, rashes, discoloration, and scarring^17^. Additionally, they compromise the skin’s immunity against bacterial and fungal infections, hinder Purkinje cell dendrite growth, disrupt DNA and RNA functions, and contribute to renal and neurological dysfunctions, cataracts, glaucoma, and Cushing syndrome^10–12,14,32^. Similarly, addition of heavy metals to cosmetics has been reported to impair fetal development in pregnant women^24,33^. Prolonged skin exposure to mercury may damage vital body organs^11,34^. Mercury exists not only in its elemental form but also as organic and inorganic compounds^35^. Mercury salts are routinely added to cosmetic products for skin-lightening purposes, despite their adverse effects. Mercury salts have been reported to irreversibly inhibit tyrosinase activity (a key catalyst in the production of melanin), which results in a decrease in pigmentation^11,12,36^. Mercury salts have a significant risk of causing gastrointestinal problems, cancer, hepatotoxicity, nephrotoxicity, and damage to the central nervous system (CNS)^11,13,36^. Notably, a study by Weldon et al*.* (2000)^37^found that 87% of 119 Mexican women using mercury containing cosmetics had elevated mercury concentrations in their urine, associated with mercury poisoning at concentrations exceeding 20 µg/L. Also, Al-Saleh et al. (2005)^38^ found elevated levels of Hg in cosmetics from many countries, with samples from Thailand, Lebanon, and England having the highest amounts ranging from 1281 to 5650 ppm. Uram et al. (2010)^39^ observed irregular labels and descriptions on the cosmetic products made in Mexico. The maximum permissible limit for Hg set by the US FDA and WHO in cosmetics is1 µg/g^13,40^.

Further, chronic exposure to arsenic, a prevalent contaminant in beauty products, causes skin problems, nervous system abnormalities, and various cancers^11,41^. Arsenic interferes with cell functions by inhibiting enzymes containing sulfhydryl groups^19^. For example, it inhibits pyruvate dehydrogenase, an important enzyme in oxidative phosphorylation, resulting in cell damage^11,19^. Its ability to induce oxidative stress and disrupt mitochondrial function contributes to neurotoxicity^11,14^. Arsenic crosses the placenta and causes spontaneous abortion during early gestation, stillbirth, preterm birth, and low birth weight^33^.

The previous studies conducted in Nigeria reported varying amounts of harmful chemicals in cosmetic products^4,5,7,42–45^. While some studies in Nigeria assessed individual components like hydroquinone or heavy metals separately (Table 1), our study examines both hydroquinone and two significant heavy metals commonly found in skin-lightening cosmetics. Additionally, to the best of our knowledge, no study has previously examined hydroquinone, mercury, and arsenic levels in skin-lightening cosmetics in Ilorin, Nigeria. To mitigate health hazards associated with prolonged exposure to these chemicals, regular assessment of their presence in skin-lightening cosmetics sold in Nigeria is imperative. Our study aims to evaluate the quantities of hydroquinone, Hg, and As in cosmetic products, shedding light on the extent of this issue in Ilorin, Nigeria. The findings will contribute relevant, and current information that can guide the implementation of policies and regulations to address this concern in Nigeria. Furthermore, it will raise awareness about the risks associated with the unrestricted use of skin-lightening cosmetics in Nigeria.

**Table 1:** Summary of previous studies on cosmetics in Nigeria.

S/N	References	Location in Nigeria	Constituents investigated	Comments
1	Egbi and Kasia (2021)^1^	Wilberforce, Bayelsa State	Study among female medical students, NDU, Nigeria	40.9% of 110 respondents use skin lightening products
2	Ajaezi et al*.* (2018)^2^	Port Harcourt, Rivers State	Arsenic, lead, mercury, cadmium, and nickel	Metal concentrations in the cosmetics ranged from 0.001–0.0161, 0.289–2.873, 0.001–0.0014, 0.001–0.334, and 0.007–2.748 mg/kg for arsenic, lead, mercury, cadmium, and nickel, respectively
3	Nduka et al*.* (2016)^3^	Onitsha, Anambra State	Hydroquinone, steroids, nitrite, N-nitrosamines, mercury, and arsenic	2 cosmetics contained mercury, 3 contained arsenic, 2 contained steroids, 9 and 11 cosmetics contained nitrites and N-nitrosamines, respectively, and 10 contained hydroquinone. Hydroquinone contents range from 1.14 to 1.83 mg/kg
4	Nduka, et al (2015)^4^	Onitsha, Anambra State	Lead, cadmium, manganese, nickel, chromium, mercury, and arsenic	61.91% of the cosmetics contained lead, mercury, and arsenic. The concentration ranges for mercury and arsenic were 0.003–0.07 and 0.002–0.005 mg/kg, respectively
5	Nasirudeen and Amaechi (2015)^5^	Kaduna, Kaduna State	Lead, cadmium, mercury, and arsenic	All samples analysed contained heavy metals above the permissible limits except arsenic
6	Popoola et al*.* (2013)^6^	Umuoji, Anambra State	Calabash stone and black antimony	High contents of lead and cadmium
7	Orisakwe and Otaraku (2013)^7^	Port Harcourt, Rivers State	Lead, cadmium, nickel, chromium, and mercury	High levels of lead, cadmium, and nickel were reported
8	Adepoju-Bello et al*.* (2012)^8^	Lagos, Lagos State	Arsenic, cadmium, lead, mercury, and nickel	All the samples contained varying amounts of all the metals investigated
9	Oyedeji et al*.* (2011)^9^	Ibadan, Oyo State	Hydroquinone, lead, iron chromium, and aluminum	Reported the presence of hydroquinone in most of the cosmetics but at lower concentrations
10	Odumosu and Ekwe (2010)^10^	Jos, Plateau State	Hydroquinone	The 10 creams investigated contained hydroquinone (3 of the 10 creams had hydroquinone content above 2%)
11	Olumide et al (2008)^11^	Nigeria	Reviewed cosmetics ingredients and adverse effects	Emphasised the complications of harmful ingredients in cosmetics
12	Adebajo (2002)^12^	Lagos, Lagos state	Epidemiological study (hydroquinone, corticosteroids, mercury)	77.3% of people investigated use skin-lightening cosmetics (male = 27.6%; female = 72.4%)

## Materials and methods

### Materials

Reference hydroquinone powder (Batch No. L186301702) was purchased from Loba Chemie PVT. Ltd., India. Ten skin-lightening cosmetics were randomly purchased within the Ilorin metropolis. All other chemicals and reagents were of analytical grade.

### Equipment

Automatic micropipette (1 mL), TLC plates, electronic analytical balance (PA214, Ohus, USA), eelectric oven (OV/50/DIG-R38, Genlab), electric water bath-420 (1111-40-DFSKW-1000DB, Lab Science, England), UV lamp (254 nm), melting point apparatus (AVI, China), whatman filter paper 1 (10 mm), nanodrop photometer (C40 version: NPOS 4.2 build 14,900 serial No: S40727), buck scientific atomic absorption spectrophotometer (210/211 VGP).

### Collection of skin-lightening cosmetic samples

Ten (10) skin-lightening cosmetics were randomly purchased from different cosmetics outlets across three local government areas in Ilorin (Ilorin South, Ilorin Central, and Ilorin North), Nigeria. Virtual inspection and registration verification of all samples were conducted in compliance with WHO recommendations^46^. The samples were identified by their names, the name of the company, batch number, National Agency for Food and Drug Administration and Control (NAFDAC) registration number, manufacturing, and expiration dates, as well as the manufacturer’s address. The samples were labelled A to K and kept in a cool, dry place away from sunlight until analysis.

### Identification of reference hydroquinone powder

The reference hydroquinone powder was further identified by preparing a 100 µg/mL test solution of the powder in methanol and scanning it in a UV spectrophotometer (Nanodrop) between 200 and 400 nm to determine its maximum absorbance wavelength (λmax). The melting point of the reference hydroquinone was also determined using the AVI digital melting point apparatus.

### Identification of hydroquinone in skin-lightening cosmetic samples

#### Sample preparation

From each sample, 0.5 g was weighed using an electronic analytical balance and transferred into a 250 mL beaker. Absolute methanol (25 mL) was then added. The mixture was homogenised for 5 min in a water bath at 60 °C and allowed to cool until the fat content separated. Whatman filter paper 1 (10 mm) was used to separate the dissolved hydroquinone from the residues. Thereafter, the filtrate was concentrated in the oven at 60 °C, and the concentrate was used for the TLC screening of the samples for the presence of hydroquinone.

#### Preparation of solutions of reference hydroquinone powder

0.1 g of hydroquinone was weighed with an electronic analytical balance, transferred into a 25 mL beaker, and dissolved in 5 mL of methanol. The solution was used within 24 h to prevent possible degradation.

#### TLC analysis for hydroquinone in skin-lightening cosmetic samples

The TLC plates were placed in the oven at 60 °C for 1 h. The activated TLC plates were spotted with the reference hydroquinone solution, and the concentrate was obtained from each sample using capillary tubes and labelled appropriately. The spotting was done at a baseline of 1 cm from the lower end of the TLC plate, and the plate was allowed to dry. The solvent system (n-hexane/acetone, 60:40) was prepared and poured into the chromatographic tank to a depth of 0.5 cm. The tank was covered with a lid for an hour to allow saturation of the tank with the solvent. The spotted TLC plate was carefully inserted into the saturated chromatographic tank using a pair of forceps and then covered to allow for the development of the plate via an ascending technique of the solvent.

The TLC plate was removed from the tank when the solvent font reached 10 cm away from the spotted samples, and the solvent font was marked. The developed chromatogram was allowed to dry. After drying, the chromatogram was examined for possible spots using a UV lamp (254 nm), and the identified spots on the chromatogram were carefully marked. The R_f_ values were calculated for each spot of the sample using the equation below and compared with the R_f_ for reference hydroquinone. Samples with a similar R_f_ value to hydroquinone were identified and reported to contain hydroquinone.

R_f_ value was calculated using the formula:

R_f_ = $$\frac{\mathrm{Distance\: moved\: by\: solute}}{\mathrm{Distance\: moved\: by\: solvent\: font}}$$

### Preparation of standard stock and working standard solutions

0.1 g of the reference hydroquinone standard was accurately weighed using an electronic analytical balance and transferred into 100-mL volumetric flasks. It was then dissolved in 10 mL of methanol to obtain a standard stock solution of 1000 µg/mL (the primary standard solution). From the primary standard solution, a 0.5 mL aliquot was pipetted out and made up to 5 mL to obtain a secondary or working standard solution of 100 µg/mL.

### Generation of the standard calibration curve

From the secondary standard solution, aliquots of 0.25, 0.5, 0.6, 0.75, 1, 1.25, 1.5, 1.75, 2, 2.25, and 2.5 mL were pipetted and transferred to a series of pre-labelled test tubes and made up to the 5 mL mark with methanol to obtain a 5, 10, 12, 15, 20, 25, 30, 35, 40, 45, and 50 µg/mL calibration standard. Each of the concentration levels was prepared in triplicate, and the absorbances were read at 290 nm on a Nanodrop UV Spectrophotometer and recorded accordingly. The standard calibration curve was plotted on Excel using the average absorbances of the triplicate measures (n = 3) against corresponding concentrations.

### Sensitivity

The limit of detection (LOD) and the limit of quantification (LOQ) were determined according to the International Council for Harmonization (ICH) guidelines^47^. LOD and LOQ for hydroquinone were calculated using 3.3*σ/*S* and 10*σ/S respectively, where σ is the standard deviation of the y-intercept and S is the slope of the calibration curve.

### Method validation

The method was validated according to ICH guidelines for validation of analytical procedures (ICHAS)^47^. Typical validation characteristics evaluated include linearity, range, precision, and accuracy.

### Intra-day and inter-day precision

Intra- and inter-day precision was performed by analysing three different batches of the test solutions, each prepared in triplicate, within 24 h (8 a.m., noon, and 4 p.m.) and on three consecutive days, respectively. Observed mean concentrations, standard deviations, % relative standard deviation (%RSD), and % recovery were calculated.

### Assay of hydroquinone in skin-lightening cosmetic samples

This study adopted and validated the previously reported assay method for hydroquinone in cosmetic samples^42,45^. 0.5 g of each was weighed with an electronic analytical balance and transferred to a 250-mL beaker, to which 25 mL of methanol was added. The mixture was homogenised for 5 min in a water bath at 60 °C and allowed to cool until fat separation occurred. The solution was then filtered using Whatman Paper 1 (10 nm). 0.1 mL of the filtrate was pipetted out using an automatic micropipette and transferred into the well-labeled test tubes. This was then made up to a 10 mL mark with absolute methanol, properly mixed, and the absorbance read at 290 nm on a nanodrop UV spectrophotometer. This procedure was done in triplicate (n = 3) for each sample. The mean absorbance reading for each sample was extrapolated from a freshly prepared calibration curve to obtain the concentration of hydroquinone present in each sample.

### Determination of mercury (Hg) and arsenic (As) in skin-lightening cosmetic samples

#### Sample preparation, digestion, and analysis

First, 0.5 g of each cosmetic sample was weighed in duplicate and transferred into a cleaned, dried 25-mL beaker. A 10 mL solvent mixture of concentrated nitric acid and perchloric acid (2:1) was added to each sample and used for digestion. The beaker was covered with a coverslip and placed on a hot plate at a regulated temperature of 105 °C under a fume cupboard, and then heated until there was a colour change from black or brown to colourless to complete the digestion process. The digested sample was allowed to cool, and the clear digest was filtered through Whatman filter paper into a cleaned, dried, 25-mL volumetric flask, and 5 mL of concentrated HCl was added to stabilise the metals. The resultant solution was made up to the 25-mL mark with de-ionized water. The same procedure was repeated for blank reagents without samples. This final solution was kept for the assay of mercury using a cold vapour atomic absorption spectrophotometer model at 253.7 nm with deuterium background correction to determine the amount of mercury in the sample.

Second, arsenic was analysed using Hydride Generation Atomic Absorption Spectrophotometry (HGAAS), which involves the conversion of all arsenic to As^3+^ species. 5 mL each of a 5% KI and a 1 M HCl solution was added to a 5 mL aliquot of the digested sample in a cleaned, dried, 25 mL volumetric flask, and the volume was then made up to the 25 mL mark with de-ionized water. The solution was allowed to stand overnight at room temperature for complete conversion of As^5+^ to As^3+^, which was then analysed using HGAAS for total arsenic content at 193.7 nm with deuterium background correction. The procedure was repeated with blank reagents without a sample. The amounts of mercury and arsenic present in each sample were calculated in mg/g using the formula presented below:

$$\mathrm{Amount\: of\:mercury\: or\: arsenic }(\mathrm{mg}/\mathrm{g})=\frac{\mathrm{Concentration}\:\mathrm{ of\: Hg \:or\: As\: in\: ppm\: X\: Volume\: of\: digest}}{\mathrm{Sample\: weight }(\mathrm{g})}$$

#### Quality control for the assays

Quality assurance and quality control procedures were used to ensure the high quality of the analytical data. The solutions of reference standards for mercury and arsenic (1000 mg/L) were diluted in the range of 1 to 4 mg/L. These solutions were used to prepare the AAS calibration curves for mercury and arsenic used to determine the concentrations of Hg and As in the samples. The concentrations of Hg and As in each sample were determined using a cold vapour atomic absorption spectrophotometer and Hydride Generation Atomic Absorption Spectrophotometry (HGAAS), respectively. The sensitivity (LOD and LOQ) of the methods for Hg and As estimation was checked by spiking the blank matrix at 0.5 (50%), 1.0 (100%), and 1.5 (150%) times the set limit of 1 ppm. A blank reagent was run in between samples to minimise contamination. The preparation of solutions and analyses were done in a spotless laboratory setting. The glassware that was used throughout the analyses was first carefully washed with detergent, soaked in 10% nitric acid for 24 h, rinsed with de-ionized water, and dried in an oven. All the reagents used were of analytical grade.

### Statistical analysis

The statistical analysis of the results was performed on Microsoft Excel 2016 (Microsoft Inc., Office 2016). The data obtained were presented as mean, standard deviation (SD), relative standard deviation (RSD), and regression coefficient (r^2^).

## Result and discussion

**Figure 1:**
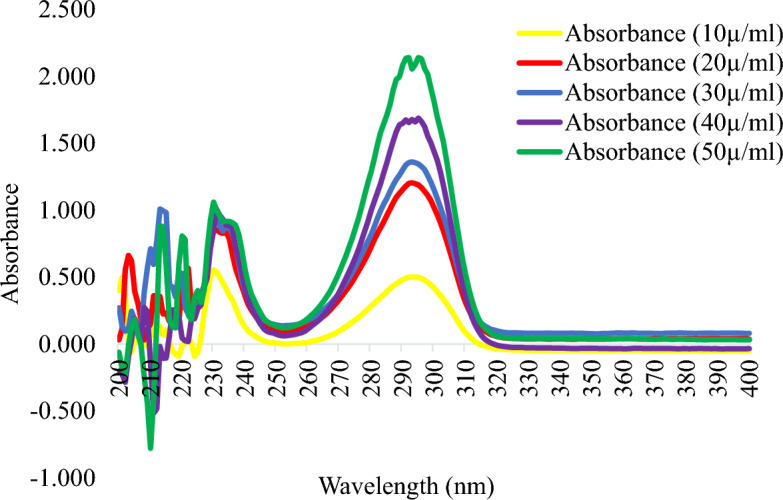
Spectra of standard hydroquinone powder between 200 and 400 nm for five different concentrations show maximum absorbance at 290 nm.

**Figure 2:**
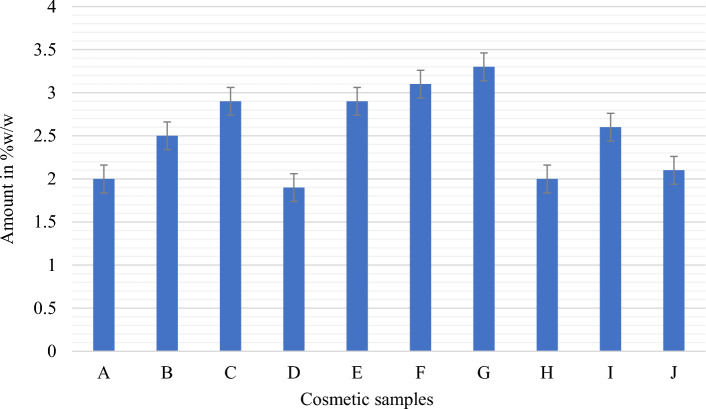
Percentage composition of hydroquinone in cosmetic samples A-J using an ultraviolet spectrophotometer at 290 nm.

**Figure 3:**
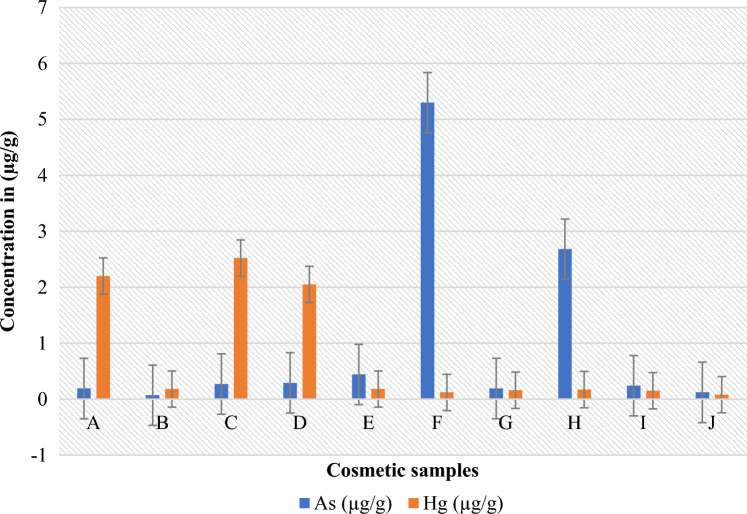
Mercury and arsenic levels in µg/gin skin-lightening cosmetics measured using AAS at wavelengths of 253.7 and 193.7 nm, respectively.

This study demonstrated that the cosmetic products that were randomly selected for evaluation had significant amounts of hydroquinone, mercury, and arsenic. Table 2 lists the specific characteristics of the skin-lightening cosmetics that were the subject of this investigation, whereas Table 3 lists the optical characteristics of the calibration curve for the UV spectrophotometer. The spectra patterns for the reference hydroquinone powder at different concentrations, ranging from 10 to 50 µg/mL, are shown in Figure 1. Figure 2 displays the samples with hydroquinone concentrations that are above the permissible limit for cosmetic products. The precision for the analysis of hydroquinone is shown in Table 4. Table 5 shows the optical characteristics of mercury and arsenic as well as the AAS instrumentation parameters. While the levels of arsenic in samples F and H were extremely high, samples A, C, and D showed significant amounts of mercury (Hg) in the cosmetic samples, which are over the permitted limit of 1 µg/g for cosmetic products (Figure 3).

**Table 2:** Descriptive characteristics of the skin-lightening cosmetic samples.

Sample	Batch No	Mfg/Expiry Date	NAFDAC Reg No	Country of origin	Composition (claims on product pack)
A	6156000163260	24-11-2020/24-11-2023	A2-1231	Not on the label	Stearic acid, c16–c18, petroleum jelly, mineral oil, cetiol, BHT, citric acid, parabens, vit. C, sodium sulfite, fragrance, coconut oil, carrot oil, ethyl diglycol, sorbitol, kojic acid, HN, white demineralised water
B	6181100536485	12-2020/12-2022	Not on the label	Cote d’ Ivoire	Water, stearylic alcohol, stearic acid, petroleum jelly, white oil, IPM, glycerin, propylene glycol, kojic acid, AHA, sodium metabisulfite, citric acid, fragrance, papaya extract
C	6028050090257	21-11-2020/11-2025	Not on the label	Togo	Vaseline oil, petroleum jelly, aqua, stearic acid, vitamin E, AHA, silicone oil, BHT, lorol c18, isopropyl myristate, fragrance
D	6182000100486	08-2020/08-2023	02-6454	Cote d’ Ivoire	Water(aqua), mineral oil, petroleum (paraffin), lanolin, stearic acid, cetyl—stearylic alcohol, isoprpyl myristate, BHT, methyl paraben, propyl paraben, sodium lauryl sulfate, sodium sulfite, citric acid, glycerin, tocopheryl acetate (vit E), fragrance, hydroquinone (2%), collagen, carrot oil
E	6181100530025	24-11-2020/24-11-2023	02-6822	Cote d’ Ivoire	Aqua (Eau), petroleum, mineral oil, stearic acid, cetearyl alcohol, isoprpyl myristate, glycerin, hydroquinone (2%), fragrance, methyl and propyl paraben, carrot oil, vit E (0.2%), sodium meta bisulfite
F	Not on the label	12-2020/12-2022	Not on the label	Cote d’ Ivoire	Acid stearic, Vaseline, vitamin E, cetremide, glycerine, MPG, conservateur antioxidant, hydroquinone, carotene 0.1%
G	6181100287943	10-12-2019/19-12-2022	02-6822	Cote d’ Ivoire, Abijan	Petrolatum, stearic acid, cetearyl alcohol, mineral oil, isopropyl myristate, propylene glycol, glycerin, methyl and propyl paraben, sodium metabisulfite, sodium lauryl sulfate, vitamin C, vitamin E, allantoin, aqua (water), fragrance
H	6186000199006	09-2018/09-2022	02-6454	Togo—Zone Franche	Mineral oil, propyl glycol, vit E, water (aqua), glycerin, white, parabens, fatty alcohol, petroleum jelly, stearic acid, sodium sulfite, collagen, silicon fluid, BHT, lanolin, fragrance
I	6181100320244	122019/12-2021	02-1475	Not on the label	Stearic acid, petrolatum, mineral oil, isopropyl myristate, stearyl alcohol, glycerin, propylene glycol, cetrimonium bromide, methyl paraben, propyl paraben, tovopheryl acetate (vit E), hydroquinone (2%), sodium metabiisulfite, allantoin, *Daucus caritona*, *Simmondsia chinensis* (jojoba), seed oil, aqua (water), fragrance
J	6181100283051	022019/02-2023	Not on the label	Togo–Lome	Demineralised water, hydroxide, acid alpha, petroleum (paraffin), mineral oil, kojic acid, isopropyl myristate, fragrance, *Simondsia, chenensis* (jojoba oil), triticum vulgare (wheat germ oil), tocopheryl acetate (vit E), ascorbic acid (vit C), white up, sodium sulfite, triethanolamine UV protection

**Table 3:** Optical characteristics of the calibration curve for UV spectrophotometers.

Parameters	Results
Detection wavelength (λ_max_) Linearity range Regression equation Correlation coefficient (r^2^) Limit of detection (LOD) Limit of quantification (LOQ)	290 nm 5–50 µg/mL 0.0307x + 0.02 0.9993 0.75 µg/mL 2.28 µg/mL

Abbreviation: λ_max_-wavelength of maximum absorption.

**Table 4:** Precision, intraday, and inter-day validation for the analysis of hydroquinone.

Parameter		Settings	Concentration (SD) µg/mL	%RSD	%Recovery
Precision					
Intraday	Time (hr)	0 4 8	19.65 (0.03) 20.96 (0.02) 20.49 (0.03)	0.17 0.11 0.13	98.23 104.82 102.47
Inter-day	Time (hr)	0 24 48	19.97 (0.07) 19.72 (0.04) 19.65 (0.03)	0.35 0.19 0.17	99.85 98.62 98.23

*Abbreviations: SD − Standard Deviation (represented by values in bracket); RSD − Relative Standard Deviation. All values are means of triplicate.

**Table 5:** AAS instrumentation parameters and optical characteristics for mercury and arsenic.

Parameters	Elements	
	Mercury (Hg)	Arsenic (As)
Detection wavelength (λ_max_) (nm) Slit width (nm) Atomization temperature (^o^C) Limit of detection (LOD) µg/L Limit of quantification (LOQ) µg/L Correlation coefficient (r^2^)	253.7 0.7 300 0.5 1.65 0.9983	193.7 0.7 900 1.0 3.3 0.9991

### Descriptive characteristics of the skin-lightening cosmetic samples

All the products were within the labelled expiration date as of the time of the analysis, while only six of the ten products were registered with the National Agency for Food and Drug Administration and Control (NAFDAC). Most of the products evaluated were manufactured in Cote d'Ivoire (54.6%) and the Republic of Togo (27.3%). Two of the products investigated had no information about the country of origin. Three of the products explicitly indicated the presence of 2% hydroquinone on their product labels. Similarly, one of the cosmetics indicated the presence of hydroquinone on its label but omitted the amount.

In contrast, the remaining six products neither acknowledged the presence of hydroquinone nor disclosed any related amount on their skin-lightening cosmetic labels. Also, none of the ten skin-lightening cosmetics purchased provided any information about the presence of mercury or arsenic on their product labels. Furthermore, one sample out of the ten failed to include a batch number on its label. Surprisingly, it was observed that none of the ten skin-lightening cosmetics analysed were manufactured in Nigeria. This suggests that a significant portion of these skin-lightening products were imported into the country, potentially without adequate consideration for the possible harmful effects of their constituents.

### Identification of reference hydroquinone powder using UV scans and melting points

From Figure 1, the lowest and highest absorbance readings were obtained at concentrations of 10 and 50 µg/mL, respectively. The peak absorbance was obtained at 290 nm, which corresponds to the British Pharmacopoeia (BP) specification for hydroquinone (BP 2016). The melting point obtained was 172 °C, which also corresponds to the BP specification. Thus, the identity of our reference hydroquinone was confirmed with a UV scan and melting point determination.

### Identification of hydroquinone in the skin-lightening cosmetic samples

The result obtained shows that all ten skin-lightening cosmetic samples had comparable R_f_ values with the reference hydroquinone powder, indicating the presence of hydroquinone in the samples.

### Quantitative determination of hydroquinone in skin-lightening cosmetic samples

The concentration of hydroquinone in each cosmetic sample was determined using a validated UV-spectrophotometric method at a wavelength of 290 nm. The method showed good linearity over a concentration range of 5 to 50 µg/mL (Table 3). The linearity equation was y = 0.0307x + 0.0192, and the mean regression coefficient (r^2^) was 0.9993 (Table 3). The method showed good precision and accuracy, with percentage relative standard deviation (%RSD) and percentage (%) recovery ranges of 0.01–0.35% and 95.85–103.56%, respectively (Table 3). The results showed that all the %RSD was below 2%, thereby attesting to the sensitivity, reliability, and accuracy of the analytical method. The limits of detection (LOD) and limits of quantification (LOQ) obtained were 0.7524 and 2.2801 µg/mL, respectively (Table 3). Intra-day validation gave the lowest and highest percentage recovery of 98.23% and 104.82%, respectively. The inter-day validation gave 98.23% and 99.85% as the lowest and highest percentage recovery, respectively (Table 4).

Quantitative analysis to determine the content of hydroquinone in the ten cosmetic samples showed varying amounts, ranging from 1.9 to 3.3% w/w. Samples D and G contained the lowest and highest amounts of hydroquinone, respectively. Further, samples A, D, and H contained 2%, 1.9%, and 2% w/w hydroquinone, respectively (Fig. 2), which were within the 2% w/w maximum permissible limit set by the WHO for cosmetics^48^. The remaining seven samples (B, C, E, F, G, I, and J) were found to contain hydroquinone in amounts ranging from 2.1 to 3.3% w/w, surpassing the allowable limit of 2% for cosmetic products^48^. In this study, most of the cosmetic packages did not provide information about the presence of hydroquinone, while only four samples explicitly stated the inclusion of hydroquinone on their product labels. Among these, only sample D contained the permitted amount of hydroquinone (1.9% w/w). Conversely, samples E and I contained 2.9 and 2.6% w/w of hydroquinone, respectively, exceeding the permitted threshold for cosmetics. Despite claims on their labels that they contained 2% w/w hydroquinone, samples D, E, and I were found to contain higher levels. Similarly, sample F was assayed to contain 3.1% w/w hydroquinone, surpassing the allowable limit. Notably, although sample F indicated the presence of hydroquinone on its label, it failed to specify the amount present.

These findings unveil a concerning reality: many skin-lightening cosmetics marketed in Ilorin, Nigeria contain varying and often excessive amounts of hydroquinone. What is particularly troubling is that these hydroquinone concentrations surpass the permissible limits for cosmetics. Consistent with other studies are the facts that most skin-lightening products contain chemicals such as hydroquinone and heavy metals that make them unsafe for use^13,28^. Despite this knowledge, these products continue to be widely distributed and used without strict regulatory control. Our findings also revealed that some of these cosmetic products marketed in Ilorin, Nigeria lack NAFDAC approval. Regrettably, many of those with NAFDAC approval failed quality assessments, surpassing the permissible limits for hydroquinone in cosmetics^28,49^. Prolonged and consistent use of hydroquinone-containing cosmetics elevates the risk of developing disorders attributed to hydroquinone^4,25–27^.

Long-term use of hydroquinone in cosmetics can lead to severe and significant adverse health effects, including conditions such as carcinoma^4,9,24^ and ochronosis^21,25^. These are diseases of public health importance, underscoring the necessity to proactively prevent their occurrence. Therefore, implementing preventive measures becomes paramount, and a crucial aspect of this involves eliminating potential risk factors such as harmful substances in cosmetic products. Moreover, the ramifications of long-term hydroquinone usage extend beyond these serious disorders. Prolonged exposure to hydroquinone has been associated with a range of adverse effects, including irritative dermatitis, melanocyte destruction, contact dermatitis, neuronal damage, and mutations^4,21,26–28^.

### Mercury and arsenic contents in skin-lightening cosmetic samples

Skin-lightening cosmetics used by women, young girls, and certain men for cosmetic purposes often contain heavy metals like mercury and arsenic^50^. The present study evaluated the mercury content of ten samples of skin-lightening cosmetics. The observed mercury concentrations ranged from 0.08 to 2.52 μg/g, with the highest and lowest concentrations being detected in samples C and J, respectively (Fig. 3). In an effort to ensure consumer safety, both the FDA and WHO have set a limit of 1.0 μg/g for mercury content in skin-lightening cosmetics^13,40,49,51^. Significantly, the findings of this study revealed that each of the ten examined skin-lightening cosmetics contained varying amounts, and three of these samples exceeded the permissible limit set for cosmetic products, indicating an unsettling presence of elevated mercury levels.

Similarly, none of the cosmetic products displayed product labels indicating the presence of mercury on their packaging, corroborating the previously reported findings^4^. The exceedingly high Hg levels in some of these cosmetics raise serious health concerns^40^. The identification of excessive mercury levels in three of the examined cosmetics serves as a crucial warning of potential health risks for regular users of skin-lightening cosmetics. Additionally, it is important to note that even relatively small quantities of mercury in skin-lightening cosmetics have been shown to have harmful effects. For instance, it was discovered that mice exposed to skin-lightening cosmetics containing 0.319 μg/g of mercury had histological abnormalities in their brain, liver, and kidney tissue^38^.

Also, regular use of mercury-containing cosmetics during pregnancy and lactation was reported to cause growth abnormalities in newborns^11,52^. Further, long-term exposure to high concentrations of Hg has been associated with adverse health effects, including hepatic damage^53^, renal impairment^54–56^, and neurological damage^11^, such as ataxia, muscle weakness, increased tendon reflex, numb limbs, speech distortion, and difficulty chewing and swallowing^11^. Many other studies have also reported high mercury concentrations in skin-lightening cosmetics^38,39^. Prolonged exposure to mercury causes significant health risks of utmost public health importance. Such exposures have contributed to a heightened prevalence of cancers, neuronal disorders, and hepatic and renal failure^11,13,36^.

The severe health problems caused by exposure to Hg have prompted many countries, like the EU^29^, the US^57^, Canada^58^, and some African countries^39^(Ghana, Uganda, and Nigeria), to impose bans on the use of Hg in cosmetic products^40,57^. Nevertheless, the effects of the ban on mercury in cosmetics remain uncertain in Nigeria, given that cosmetics containing mercury are still widely consumed by users^5^. The intentional use of mercury in skin-lightening cosmetics is prohibited by the Drugs and Cosmetics Act^59^. The prevalent occurrence of unregulated hazardous chemicals and heavy metals in skin-lightening cosmetics is a well-documented issue in many developing countries. Nonetheless, this concern transcends national boundaries and is indeed a global problem. For example, Sahu et al*.* (2014)^59^ reported mercury in 17 of 32 skin-lightening cosmetics investigated. In a similar study by Hammann et al. (2014)^60^, a total of 549 skin-lightening cosmetics selected from 32 countries were confirmed to contain mercury. Addressing this menace requires the implementation of stringent regulations and effective compliance strategies to reduce the prevalence of cosmetics containing mercury. From a global perspective, it becomes imperative to establish legislation and rigorously enforce measures that prohibit the production, importation, and export of cosmetics containing mercury. Such measures would undoubtedly prove advantageous in mitigating the public health hazards associated with the routine use of mercury-containing cosmetic products.

Conversely, samples B and F contained 0.07 and 5.3 µg/g of arsenic, respectively, representing the lowest and highest measured amounts (Fig. 3). When compared to the previous studies, these values stand out as significantly elevated. For instance, the amount of arsenic found in cosmetics by Adepoju-Bello et al*.* (2012)^61^ ranged from 0.006 to 0.013 µg/g, and a similar study reported a maximum of 0.2 μg/g arsenic in body lotions^62^. However, the amounts of arsenic in samples F and H of our study were 5.3 μg/g and 2.68 μg/g, respectively (Fig. 3). These amounts were too high in cosmetic products, although some previous studies also reported high amounts of arsenic in skin-lightening cosmetics. For example, Mohammed et al*.* (2017)^18^ reported a range of 1.016–6.612 μg/g of arsenic in skin-lightening cosmetics, while Alquadami et al*.* (2013)^63^ reported arsenic levels between 0.34 and 14.76 μg/g in 34 examined skin-lightening cosmetics.

It is noteworthy that despite our findings of elevated arsenic levels in samples F and H (Fig. 3), the product labels on the cosmetic packaging did not mention arsenic as one of the ingredients. Furthermore, our study identified arsenic in all ten skin-lightening cosmetics studied. This raises questions regarding the source of arsenic in cosmetics, which could stem from intentional inclusion or inadvertent contamination during the manufacturing process. The introduction of arsenic into cosmetics could potentially occur through the tear and wear of metallic equipment and machinery used during the manufacturing processes^64^. However, it is important to note that significant arsenic contamination through these processes is not typically anticipated to be substantial.

Chronic exposure to arsenic has been reported as a significant risk factor for developing various types of cancer^11,65–67^. Long-term exposure to arsenic has been associated with a range of health problems. These encompass palmar and solar keratosis, gastrointestinal symptoms such as vomiting and diarrhea, dermatological abnormalities, neuropathy, ischemic heart disease, cognitive impairment including confusion and memory loss, respiratory disorders, hormonal imbalance, compromised immune system function, and an increased risk of diabetes^64,66,67^. The ability of arsenic to induce oxidative stress and mitochondrial dysfunction is the main cause of the observed neurological diseases^11^. Oxidative stress damages the DNA, which results in neuronal cell death^14^. Despite the harmful effects of arsenic, it has been found to be a component of eye shadow, lotions, cosmetics, and lipsticks^68^. Regrettably, no strict regulations prohibit the use of arsenic in cosmetic preparations except in the European Union^69^. Hence, there is currently no established limit to the amounts of arsenic that are permitted in cosmetics. This regulatory gap underscores the urgent need for comprehensive guidelines to restrict and monitor the presence of arsenic in cosmetic preparations to mitigate potential health hazards associated with its usage.

The health hazards and public health relevance of undue exposure to hydroquinone, mercury, and arsenic require that policymakers and regulatory authorities put strict restrictions on the production and importation of skin-lightening cosmetics containing these dangerous chemicals. Such measures are crucial in curbing the proliferation of unregulated cosmetic products in the market, thereby ensuring the well-being of Nigerian citizens, and mitigating the economic hardship brought about by the burden of associated health problems.

## Conclusion

The ten cosmetic products examined in this study contained hydroquinone, mercury, and arsenic in varying amounts. Based on our findings and the previously reported studies, it is obvious that skin-lightening cosmetics containing these dangerous chemicals remain readily accessible in Nigeria. This persists despite the associated health risks and the explicit prohibition of their production, importation, and distribution. As a critical measure, it becomes imperative to declare the use of these products illegal, subjecting them to stringent regulatory oversight guided by the stakeholders entrusted with ensuring the safety of goods and services in Nigeria. Therefore, the establishment and enforcement of comprehensive guidelines governing the manufacturing and importation of cosmetic products in the country are imperative. This framework should ensure a thorough safety evaluation of both the raw materials and the final products prior to their introduction to the market. To protect the health of Nigerians, the findings in our study recommend the introduction and consistent enforcement of regular quality control assessments, overseen by relevant regulatory bodies. Such assessments should encompass skin-lightening cosmetics and all other cosmetic products available to consumers. The government has the duty to ensure that manufacturers of skin-lightening cosmetics comply with the established standards and specifications governing cosmetic production in Nigeria. Furthermore, regulatory authorities should actively cultivate public awareness and diligently oversee the strict regulatory control of skin-lightening cosmetics being marketed in the country. This unified effort is instrumental in mitigating the prevailing threat and ensuring the protection of the health and well-being of Nigerians.

## Data Availability

All data used in this study are available from the corresponding author upon reasonable request.
